# Macroalgal Bloom Biomass as a Source of Bioactive Compounds and Antimicrobial Peptides

**DOI:** 10.3390/md24040136

**Published:** 2026-04-15

**Authors:** Nedeljka Rosic, Isidora Skrlin, Carol Thornber

**Affiliations:** 1 Faculty of Health, Southern Cross University, Gold Coast, QLD 4225, Australia; 2 Marine Ecology Research Centre, Southern Cross University, Lismore, NSW 2480, Australia; 3 Faculty of Science, The University of Queensland, St. Lucia, QLD 4072, Australia; i.skrlin@student.uq.edu.au; 4 School for the Environment, University of Massachusetts Boston, Boston, MA 02125, USA; carol.thornber@umb.edu

**Keywords:** macroalgae, algal bloom, algal drift, bioproducts, antimicrobial peptides, bioactivity, seaweeds, in silico screening, marine bioprospecting, omics

## Abstract

Macroalgal species are widely distributed throughout the world’s oceans and are well recognised for their biotechnological, ecological, and pharmacological potentials, containing a wide range of diverse bioactive compounds. In many coastal habitats worldwide, excessive accumulations of algal biomass (including rapidly growing blooms and drift accumulations resulting from dislodgement from benthic habitats) are commonplace and can pose environmental and economic challenges. In this study, we report occurrences of algal blooms and drift accumulations during 2024 and 2025 involving three major macroalgal clades, Chlorophyta, Phaeophyceae, and Rhodophyta, from two distinct marine regions: the North Atlantic Ocean and the South Pacific Ocean. Species identified included *Grateloupia turuturu*, *Polyides rotundus*, *Ascophyllum nodosum*, *Ulva* spp., *Sargassum* spp. and *Fucus* spp., among others. The indicated species are known for their diverse pharmacological properties, including antimicrobial, antioxidant, and anti-inflammatory effects. Specialised bioinformatic tools were employed to assess the potential of identified macroalgae as a source of antimicrobial peptides (AMPs). For selected macroalgal species, in silico screening of publicly available databases was performed to identify previously reported and characterised AMPs associated with these species. This in silico approach presents a promising strategy for discovering novel antimicrobial agents with potential activity, especially against drug-resistant bacteria. Finally, applying proteomics methodologies for in silico evaluation of the selected algal species advances modern technologies for the sustainable use of natural resources.

## 1. Introduction

Algae are a polyphyletic group, encompassing a wide range of sizes and distinct evolutionary lineages, and are found in all the world’s oceans. Both microalgae (unicellular, photosynthetic microorganisms, including diatoms, dinoflagellates, and other groups) and macroalgae (including green, red, and brown seaweeds) are found in various marine habitats, ranging from tropical to polar regions, and from intertidal to subtidal zones [1,2,3]. Multicellular macroalgae comprise a diverse array of taxa from distinct evolutionary lineages (Rhodophyta—red; Chlorophyta—green; and Phaeophyceae—brown), exhibiting diverse life history strategies including r-selected and K-selected species, isomorphic and heteromorphic life cycles, and a wide range of ecological niches [4]. While many species are benthic and grow attached to rocks or other hard surfaces, some spend part or all of their lives drifting in the pelagic zone [2,5].

Algae play important roles as primary producers, nursery habitats, and food sources within diverse marine food webs and ecosystems [6,7]. The environmental and economic benefits of algae are well documented, particularly regarding their production of valuable bioactive compounds [8,9,10,11]. The marine natural products of algal origin include a wide variety of proteins [12,13], lipids [14,15], polysaccharides [16,17], pigments and UV filters such as Mycosporine-Like Amino Acids (MAAs) [13,14,15,16,18], and vitamins [19,20,21]. The growth and physiological performance of marine species, including the production of various valuable bioactive compounds, are heavily influenced by external conditions such as water quality, nutrient availability, UV levels, and temperature changes [22,23,24,25,26,27,28].

Algal blooms are defined as the rapid proliferation of phytoplankton or macroalgae, resulting in increased cell density that may have significant negative consequences on ecosystems [29]. Blooms frequently result from large and/or rapid increases in algal biomass due to favourable physical factors, including available nutrients (typically as dissolved inorganic nitrogen), warmer water temperatures, and/or circulation and wind patterns that concentrate biomass in specific regions [30,31]. Macroalgal blooms occur when drifting macroalgae accumulate in large quantities of one or more species due to eutrophication [32,33]. Much research has focused on the causes and impacts of these blooms [34,35,36]. When these blooms negatively impact nearshore environments, resulting in hypoxic conditions that lead to the death of fish, negatively impacting the marine ecosystem and fouling of coastal embayments, it leads to a decline in species abundance [37,38,39]. While some algal blooms are sporadic in nature, many are predictable, occurring in the same areas repeatedly from one year to the next [40,41].

Although macroalgal blooms can have serious negative consequences for the environment, the abundant biomass produced may also have unexpected positive impacts [29,42]. Depending upon the species composition of a particular bloom, the abundant biomass may have a range of human uses. Many macroalgal species synthesise secondary metabolites, including antimicrobial compounds such as polysaccharides, phenols, terpenes, and fatty acids [43,44,45]. UV-absorbing compounds such as MAAs exhibit multiple bioactivities, including antioxidative, anti-inflammatory, and anti-ageing effects, with significant utility across a range of environmental and biopharmaceutical applications [46,47,48,49]. Modern omics tools including proteomics are increasingly used for the discovery of novel natural products, improving our understanding of their innovative applications in medicine, cosmetics and other industries [50,51,52,53].

In recent years, interest in antimicrobial peptides (AMPs) has increased as a potential alternative to conventional antibiotics [54,55] due to the challenges posed by antimicrobial-resistant (AMR) bacteria to current global health treatments, particularly in the post-pandemic era [56,57,58]. AMPs are small peptides, usually shorter than 100 amino acids and found in prokaryotic and eukaryotic organisms, that are evolutionarily conserved and important in immune defence [59,60]. The majority of AMPs are cationic, containing both hydrophobic and hydrophilic residues, and exhibiting amphiphilic structures that allow them to bind and interact with the membranes of microbial pathogens, leading to their destruction [60]. This effect on the bacterial membrane is one of the mechanisms of AMP actions, while the second common mode of action includes inhibition of metabolic processes related to DNA, RNA, and protein synthesis [61]. Worldwide, microbial infections are causing millions of global deaths [44] and are predicted to surpass cancer and cardiovascular illnesses as the leading cause of mortality in the near future [62]. As bacteria develop resistance to AMPs at a significantly slower rate [54], AMPs present promising alternatives to traditional antibiotic therapies [63]. Additional applications of AMPs include the use in dermatology for promoting healing processes and improving skin health [64], in urinary tract infections, and as potential diagnostic markers [65].

Here, we explored the diversity of species within two algal bloom events reported in Australia and the USA. Specific algal bloom events were targeted due to their ecological relevance, documented occurrences, and direct impact on coastal communities. In addition, local regions provided reliable access to biomass and associated data, enabling a detailed assessment of species diversity. The biotechnological potential of algal biomass was evaluated as a source of useful bioactive compounds. In addition, we assessed potential peptide combinations from the dbAMP database, which contains sequence information, functional activity data, physicochemical attributes, and structural annotations for over 35,000 peptides [66]. Finally, we also evaluated, using modern proteomics tools, the potential of the obtained biomass as a source of AMR peptides, contributing to addressing the emerging crisis of antimicrobial resistance.

## 2. Results and Discussion

### 2.1. Algal Bloom Events

**Figure 1:**
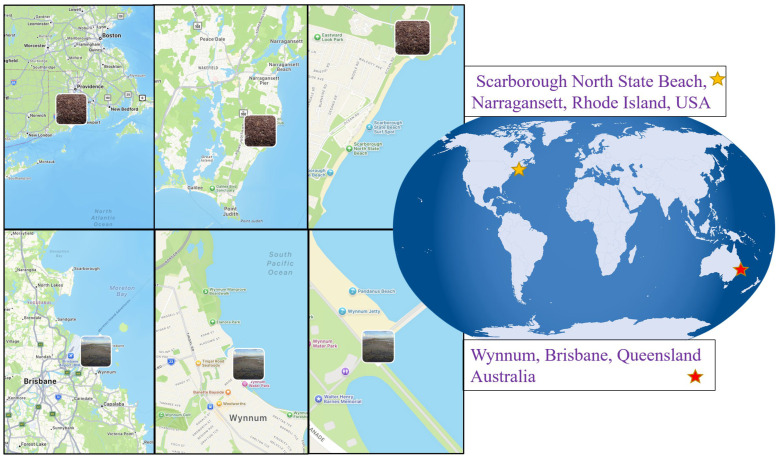
Sampling sites of algal bloom aggregations in the USA and Australia.

Two distant occurrences of algal blooms involved three major macroalgal clades, Chlorophyta, Phaeophyceae, and Rhodophyta, from two distinct marine regions, the North Atlantic Ocean and the South Pacific Ocean, in the USA and Australia (Figure 1). Documented events included both nearshore bloom formations and large offshore drift assemblages, likely reflecting variations in oceanographic conditions, nutrient regimes, and temperature anomalies observed during the study period. These algal bloom phenomena were often reported in coastal zones characterised by seasonal temperature variability, which has been recognised as a dominant factor increasing the risk of algal blooms, while variation in inorganic nutrient profiles may be useful for short-term algal bloom prediction [67,68].

**Figure 2:**
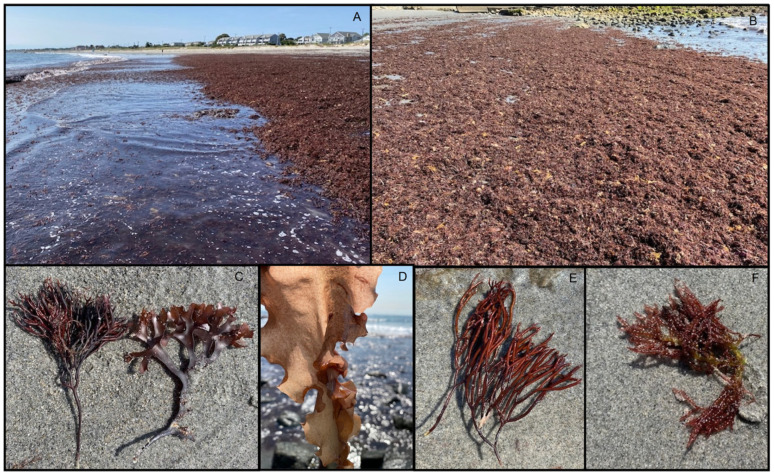
Scarborough North State Beach, Narragansett, RI, USA. (**A**,**B**) Photographs of dense macroalgal accumulations at the northern end of the beach, where these aggregations are commonly found. (**C**) The red alga *Chondrus crispus*. (**D**) The red alga *Grateloupia turuturu*. (**E**) The red alga *Polyides rotundus*. (**F**) The red alga *Champia parvula*.

Algal bloom events in the USA were commonly observed at Scarborough North State Beach (Rhode Island) during the late summer seasons and were characterised by a mix of macroalgal species, including representatives of the green, brown, and red macroalgal groups (Figure 1 and Figure 2). The algal bloom event occurred in slow-moving waters, with large algal biomass accumulating along the shore. The mean maximum temperature was 28 °C, the mean minimum temperature was 18 °C, the average rainfall was 86 mm, and the UV radiation moderate (Table 1). In August 2024, Rhode Island received above-average rainfall, exceeding the usual ~96 mm (https://www.ncei.noaa.gov/access/monitoring/monthly-report/national/202409, accessed on 27 January 2026). In other areas, heavy rain increased runoff from local agriculture, urban areas and sewage, and higher nutrient levels led to algal blooms [69,70].

In Australia, along the Wynnum foreshore in Brisbane, Queensland, algal blooms, specifically including seaweeds and blue-green algae/cyanobacteria, occur seasonally, especially during the warmer summer months from November to February [71]. In southeast Queensland, during the summer season, days are warm and humid, with average high temperatures between 27–31°C in February 2025 [72].

External conditions in Brisbane during the algal bloom event in January–February 2025 were characterised by higher total rainfall (https://www.bom.gov.au/climate/current/month/qld/archive/202501.brisbane.shtml, accessed on 27 January 2026), with total rainfall at 127% of the long-term average (Table 1). The mean daily maximum temperature was 0.3 °C above the long-term average, while the mean daily minimum temperature was 0.6 °C below the long-term average, with January and February being the warmest months for sea surface temperatures in Queensland.

### 2.2. Algal Bloom Species

Species or genus representatives identified among the bloom- and drift-forming assemblages included a mix of red, brown and green algae (Table 1). Among Rhodophyta, *Grateloupia turuturu* Yamada, *Polyides rotundus* (Hudson) Gaillon, *Chondrus crispus* Stackhouse, *Dasysiphonia japonica* (Yendo) H.-S.Kim, *Champia parvula* (C.Agardh) Harvey, *Dasya baillouviana* (S.G.Gmelin) Montagne and *Gracilaria* species were reported. Biotechnologically important species included *G. turuturu* (previously *G. doryphora*), an invasive intertidal red alga recognised for its biotechnological potential, with a broad spectrum of antimicrobial activity [73] and UV-absorbing MAA compounds [74,75]. *P. rotundus* and other red algae have been used as a valuable source of polysaccharides such as carrageenan, which is not only important in the food industry as a thickener [76], but also as an anticoagulant [77]. The red algal genus *Gracilaria* has representatives associated with algal blooms reported in Asia, the USA, and Europe [78,79,80,81,82]. *Gracilaria* species are also cultivated in aquacultures and used for agar production and human consumption [83]. *C. crispus,* an edible seaweed also known as Irish moss, has been used as a model species in research and, as a result, more data on its bioactivities, including anti-inflammatory, antioxidant, anticancer, antivenom, and antimicrobial activities, have been published [84]. The cell wall of *C. crispus* contains carrageenan, which is commercially used as a thickener [85]. Other biotechnologically significant algae include *D. japonica*, an invasive bloom-forming species that has lower levels of MAAs than some other red algae [86].

For Phaeophyceae, species reported during algal blooms included *Fucus* spp., *Sargassum* spp. and *Ascophyllum nodosum* (Linnaeus) Le Jolis, while for Chlorophyta, there were *Ulva* spp. (blade morphology), *Ulva* spp. (tube morphology), *Codium fragile* subsp. *tomentosoides* (Van Goor) P.C. Silva and *Bryopsis* spp. representatives. *C. fragile* is one highly invasive species with potential for various biotechnological applications. Extracts from this green alga were used in folk medicine to treat urinary tract disorders by stimulating the immune response via mitogen-activated protein kinases and other pathways [87]. The anti-inflammatory effect was therapeutic for the treatment of atopic dermatitis [88]. *Ulva* species (known as sea lettuce) have been recognised for their use in the food industry for both humans and animals [89] and as a source of a sustainable biomaterial applicable in industry and pharmacological compounds such as sulphated polysaccharide ulvan, characterised by immunomodulatory, antimicrobial, and anticoagulant activities [90]. Green algal species in the genus *Bryopsis* have been reported to exhibit bioactivity, including as biostimulants and biopesticides in drug discovery, and anticancer activity [91].

### 2.3. Biotechnological Applications of Algal Bloom Species

Beyond their ecological significance, the identified algal species possess a wide range of pharmacological properties (Table 2). Extracts derived from these macroalgae have shown antimicrobial, antioxidant, antiviral, and anti-inflammatory activities in both in vitro and in vivo studies (Table 2). Bioactive compounds such as sulphated polysaccharides (e.g., carrageenans, ulvans and fucoidans), polyphenols, terpenoids, and photosynthetic pigments have attracted growing interest for their potential applications in nutraceuticals, functional foods, and pharmaceutical development [8,92,93]. Therefore, although large-scale blooms and drift events may create ecological and socioeconomic challenges, they also represent underutilised biomass resources with considerable biotechnological value.

Red algae exhibit the highest protein content among macroalgae (up to 47% dry weight) and are rich in essential amino acids [109]. Rhodophyta also contain bioactive compounds, such as AMPs, which are potential alternatives to traditionally used antibiotics for drug-resistant bacterial infections [80], and MAAs, which are among the most promising natural UV-absorbing compounds [106,110,111,112,113,114]. AMPs are small molecules (commonly 6–100 amino acids) that play an important role in host innate immunity against a range of pathogenic microorganisms, including Gram-positive and Gram-negative bacteria [115,116]. These peptides act both directly, by eliminating invading pathogens, and indirectly, by modulating the host immune system, via activation of adaptive immune responses (e.g., stimulation of cytotoxic T cells) and enhancement of innate immune mechanisms involving leukocytes, such as neutrophils, as well as synthesis of cytokines and chemokines to initiate phagocytosis to remove pathogenic bacteria [117]. Studies on the structure–activity relationship have revealed that net charge, hydrophobicity, and amphipathicity are the most important physicochemical and structural determinants for AMPs’ antimicrobial potency and cell selectivity. Macroalgal AMPs reported for Irish moss, *C. crispus* [118,119], were further assessed, including their physical and chemical properties (Table 2), in silico analyses, and some combined in vitro confirmatory analyses. The AMP activity is influenced by physical and chemical properties, including peptide length, net charge, and hydrophobicity [117,120]. AMPs that are more hydrophobic, with non-polar amino acids (e.g., Leu, Ile, Val, Phe, Trp) and with some positively charged residues (e.g., Lys, Arg, His), and a net of positive charge (+2 to +9), result in enhanced peptide binding to the negatively charged bacterial member [121]. These peptides, which have a higher proportion of hydrophobic residues, act by embedding in the bacterial membrane, disrupting it and causing membrane leakage, leading to bacterial death [122,123]. A higher aliphatic index has been linked to increased AMP thermal stability, due to a higher content of specific amino acids (e.g., alanine, isoleucine, leucine, and valine), resulting in improved binding actions on bacterial membranes [61]. There are still multiple challenges with the utilisation of AMPs, including instability, potential cell toxicity, and high production costs [124]. To overcome these challenges, protein engineering [98,99,100,101] has been applied to create new proteins and peptides that optimise the use of AMPs, from sequence to functionality, against antibiotic-resistant bacteria [125].

### 2.4. Antimicrobial Peptides from Algal Blooms

For a number of macroalgae species identified during these two algal bloom events, previous research has reported a wide range of antimicrobial activities. For *Codium fragile*, the crude methanol extract (CME) and the polysaccharide portion (PSP) of the methanol extract exhibited potent antimicrobial activity against Gram-positive bacteria, *Staphylococcus aureus* and *Bacillus cereus* [126]. Crude extracts of *Ascophyllum nodosum* with the highest antimicrobial activity were found against *Staphylococcus aureus*, but not against *E. coli* [119].

A wide range of antibacterial activities was reported for extracts isolated from green macroalgae *Ulva fasciata*, with a number of bacterial species impacted, including *Enterococcus faecalis*, *Vibrio alginolyticus*, *Vibrio cholerae*, *Staphylococcus aureus*, *Salmonella typhimurium*, and *Escherichia coli* [127]; then *Aeromonas hydrophila*, *Pseudomonas fluorescens*, *Proteus* spp., *V. alginolyticus* and *Enterobacter* sp. [128]; and also *Bacillus subtilis*, *Streptococcus pyogenes*, *E. coli*, *Klebsiella pneumoniae*, *Pseudomonas aeruginosa*, *Salmonella typhimurium*, *Vibrio cholerae*, *Shigella flexneri*, *Proteus mirabilis* and *P. vulgaris* [129].

Red alga, *C. crispus* (known as Irish moss), which has been used as a model species in research, exhibits antimicrobial activity attributed to specific peptides, as demonstrated by in vitro and in silico assessments [118,130]. Within the dbAMP database (dbAMP), AMPs were found only for the model species *C. crispus*, while no information was available for other algal bloom-identified species. *C. crispus* peptides reported in the dbAMP database (Table 3) lacked in vitro-confirmed antibacterial activity [118]. Based on their physicochemical properties, *C. crispus* peptides identified in the dAMP database were positively charged (e.g., up to +9), with up to 26% positively charged residues (e.g., Arg, Lys and/or His) like what was observed in the peptide dbAMP_33037 and also with 33% hydrophobic residues (e.g., amino acids Leu, Ile, Val, Phe, Trp) such as for the dbAMP_33037 peptide. However, the optimal proportion of hydrophobic residues is usually around 50%, which is important for interactions and for disrupting the negatively charged bacterial membrane structure, while balancing overall charge and hydrophobicity is required to establish effective AMPs [131]. Regarding theoretical isoelectric points (pI), this value usually ranges between 9 and 12 or more, indicating the cationic nature of AMP in physiological pH conditions, which is needed for action against negatively charged bacterial membranes [132]. The majority of predicted AMS in Table 3, except dbAMP_33036 and dbAMP_33041, had a required high pI > 8, indicating their positive charge. The 3D structures of proposed *C. crispus* AMPs were mainly linear molecules with alpha helices present in dbAMP_33039 and dbAMP_33040 structures. The alpha-helical structures within AMPs are widely distributed in nature and are important for their effectiveness and disruptive impact on bacterial membranes, via insertion into lipid bilayers [133]. Linear AMPs often, upon interaction with bacterial membranes, change structure, forming an alpha-helix to disrupt microbial cell membranes [134,135].

Using AMP Scanner and ProtParam, physicochemical properties of potential AMPs from *C. crispus* obtained from protein hydrolysate against *S. aureus* that were not reported in the dbAMPs database were assessed in silico [136]. This included three putative AMPs, where P01 KKNVTTLAPLVF was characterised as an α-helical cationic peptide with a 0.525 Grand Average of Hydropathy (GRAVY) value, an amphipathic structure, and a +2 total charge [130]. Moreover, strong interactions were observed between the peptides identified as P07 (sequence: SAGSGNEGLSGW) and P20 (sequence: RTASSR), which interacted with bacterial enzyme DNA gyrase and membrane receptors from *S. aureus*, with strong binding energies. Bacterial DNA gyrase is a type II topoisomerase that introduces negative supercoiling into DNA, which is important for the DNA replication and transcription processes [137]. Inhibition of DNA gyrase, leading to DNA instability, inhibition of DNA replication, and cell death in bacteria and therefore, this enzyme is one of the targets of antibiotics [138]. Furthermore, negatively charged bacterial membranes are targeted by positively charged AMPs, which disrupt them by inhibiting lipid biosynthesis in Gram-negative bacteria or by blocking membrane channels and solute transport, leading to bacterial toxicity and death [139]. Further parameter analyses (ProtParam) included the instability index (II), which was 18.14 for P01 and 12.38 for P07 peptides, indicating good protein/peptide stability, whereas II for P20 was 72.53, indicating molecular instability. Others indicated that active AMPs are characterised by a stable II (below 40), a positive net charge (usually +2 to +13), and hydrophobicity [140]. The grand average of hydropathicity (GRAVY) estimates proteins’ hydrophobicity or hydrophilicity, with a positive GRAVY value indicating hydrophobic behaviour and a negative value indicating hydrophilic behaviour [141,142]. It is typically calculated using tools such as ProtParam, https://web.expasy.org/protparam/ (accessed on 29 March 2026). A positive GRAVY value was found for P01, indicating hydrophobic behaviour of this peptide, and over 50% of hydrophobic residues is consistent with effective AMPs [131]. Negative GRAVY values, as found in P07 and P20, indicate an overall hydrophilic nature of these peptides, as calculated using ProtParam. Generally, AMPs are amphiphilic, containing both hydrophobic parts (from nonpolar amino acids) that interact and disrupt negatively charged bacterial membranes and hydrophilic parts (from positively charged amino acids) that allow solubility in aqueous fluids such as blood [131]. However, only the P01 peptide was predicted to be AMP, while the other two proposed AMPs based on in vitro analyses were below the threshold for AMP prediction probability in silico (i.e., prediction probability > 0.5), indicating that in silico predictions require in vitro confirmation to meet the criteria for classification as AMPs.

AMPs produced by algal blooms are particularly promising sources for novel discoveries as they may present a unique combination of antimicrobial activities. AMPs are emerging as key tools for addressing antibiotic-resistant bacterial infections and protecting public health [56,57,58]. AMPs’ action occurs very quickly, primarily by disrupting bacterial membranes and, in some cases, by interfering with intracellular targets or modulating host immunity. Based on in vitro and in silico analyses, AMPs have demonstrated potential against Gram-positive and Gram-negative bacteria, including antibiotic-resistant strains. However, discrepancies between computational AMP predictions and experimental validation underscore the need to integrate bioinformatic screening with laboratory-based functional assays. Challenges such as peptide instability, cytotoxicity, and production cost remain barriers to translation, but advances in protein engineering and synthetic biology may offer viable pathways to optimise AMP efficacy and stability.

## 3. Materials and Methods

### 3.1. Study Area

The first study was conducted at Scarborough North State Beach, located in Narragansett, Rhode Island, 55 km from the capital city, Providence (USA). The sampling points north of the beach were located between two adjacent sites (41°23′43″ N 71°28′01″ W and 41°23′43″ N 71°27′52″ W). The sampling time was 16 September 2024, at the end of summer in the Northern Hemisphere (Figure 1 and Figure 2).

**Figure 3:**
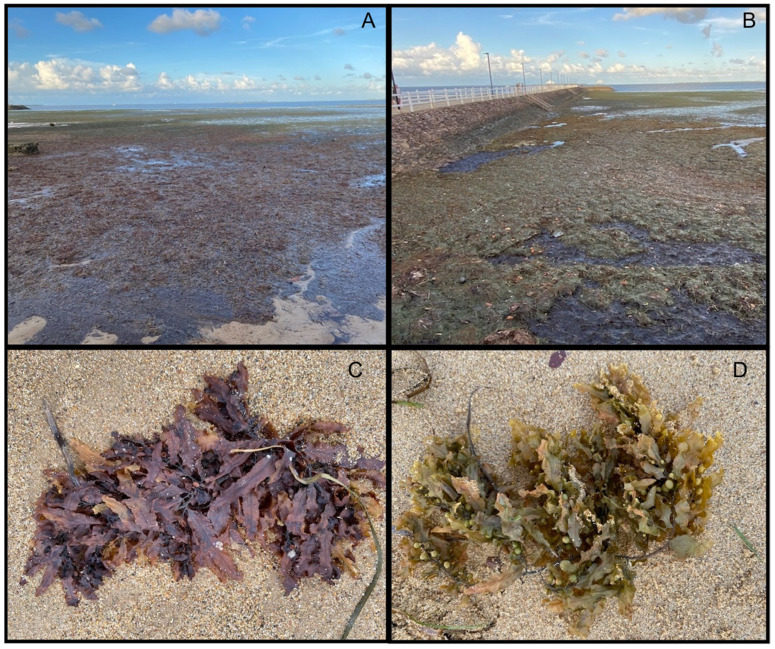
Wynnum Beach, Brisbane, Queensland (Australia). (**A**,**B**)—photographs of dense macroalgal accumulations on the beach, where these aggregations are commonly found; (**C**,**D**) *Sargassum* spp. representatives.

The second study was conducted at Wynnum Beach, in Brisbane, the capital of Queensland (Australia). The sampling points were located at the coordinates between two sites (27°26′32″ S, 153°10′41″ E and 27°26′41″ S, 153°10′52″ E). The sampling time was 1 February 2025, at the end of summer in the Southern Hemisphere (Figure 1 and Figure 3).

### 3.2. Sample Identification

At each site, abundant and dense accumulations of drift macroalgae were observed along the shoreline (USA, Figure 2; Australia, Figure 3). Both locations are known for frequent macroalgal accumulations. At each site, we collected the most common, abundant macroalgae found in the drift along the shoreline. Degraded and/or unidentifiable specimens due to significant decay were not collected. The identification of specimens to the species and/or genus level (depending on the taxon) was completed by a trained phycologist with decades of algal identification experience (C.T.) using morphological characteristics and standard taxonomic references, the Illustrated Key to the Seaweeds of New England (2nd edition) and the worldwide taxonomic database https://www.algaebase.org/ (accessed on 12 December 2025). Identified specimens were then preserved by drying using standard herbarium techniques on acid-free herbarium paper [143].

### 3.3. Abiotic Conditions Assessment

External conditions, including average temperature, rainfall, and UV level at the sites of algal blooms, were obtained from publicly available databases. The average monthly temperature in the Australian location was obtained from the Australian Government Bureau of Meteorology, along with UV indices and rainfall. The USA external conditions were obtained from the nearest meteorological station (TF Green Airport, Warwick, RI, USA). A summary of temperature and rainfall statistics for January 2025 for Brisbane and Rhode Island is presented in Table 1.

### 3.4. Identification of Antimicrobial Peptides (AMPs) Candidates

The freely available Antimicrobial Peptide Database (APD) (dbAMP, dbAMP 3.0: updated resource of antimicrobial activity and structural annotation of peptides), which provides access to over 35,000 peptides, has been used in this study [66]. The search included applying the species names identified during two algal bloom events in the AMP (dbAMP) database. Identified AMPs were further assessed using the bioinformatic tool AMP Scanner Version 2 (Antimicrobial Peptide Scanner), an open-source, deep learning-based tool designed for predicting and identifying AMPs using FASTA-formatted protein sequences (https://www.ncbi.nlm.nih.gov/genbank/fastaformat/, accessed on 12 December 2025), to predict antimicrobial activity in silico via deep learning neural network models [136]. Additional analyses included assessment of the physico-chemical properties of targeted peptides using ProtParam (https://web.expasy.org/protparam/, accessed on 29 March 2026) [115] and the ExPASy server, which provides a comprehensive bioinformatics resource (operated by the SIB Swiss Institute of Bioinformatics) [144].

## 4. Conclusions

The two geographically distant bloom and drift events documented in 2024–2025 in the North Atlantic (USA) and the South Pacific (Australia) demonstrate that macroalgal proliferations involve representatives from Rhodophyta, Chlorophyta, and Phaeophyceae groups. These natural events are not isolated phenomena; rather, they are part of global environmental change. Seasonal temperature variability, elevated rainfall, and altered nutrient regimes appear to have contributed to bloom formation and biomass accumulation in both regions, reinforcing the link between climatic anomalies and increased bloom frequency and persistence. Despite the ecological and socioeconomic challenges associated with dense shoreline algal biomass accumulations that lead to hypoxia during decay and impact tourism and fisheries, the macroalgal species identified represent a substantial and underutilised biotechnological resource. Bloom-forming species, including *Grateloupia turuturu*, *Chondrus crispus*, *Polyides rotundus*, *Ulva* spp., *Codium fragile*, *Ascophyllum nodosum*, and *Sargassum* spp., are rich sources of sulphated polysaccharides (i.e., carrageenans, ulvans, fucoidans), polyphenols, terpenoids, pigments, and UV-absorbing MAAs. These compounds exhibit antimicrobial, antiviral, antioxidant, anti-inflammatory, anticoagulant, and immunomodulatory properties with applications spanning nutraceuticals, pharmaceuticals, agriculture, and biomaterials.

Finally, a dual perspective on macroalgal blooms is highlighted: shifting oceanographic and climatic conditions have ecological consequences, and opportunistic biomass streams can be utilised due to their considerable economic and biomedical potential. Future research should prioritise (i) long-term environmental monitoring to improve bloom prediction, (ii) sustainable biomass harvesting and processing frameworks, and (iii) integrated biochemical and molecular characterisation of bloom-forming taxa to accelerate the discovery and development of high-value bioactive compounds.

## Figures and Tables

**Table 1:** External conditions at the location of the algal bloom drift in Brisbane, Wynnum Beach (Australia) in January 2025. The air temperature was measured at the nearest locations, TF Green Airport, Warwick, Rhode Island, and Scarborough North State Beach (USA), in September 2024.

Location	Mean Air Maximum Temperatures (°C)	Mean Air Minimum Temperatures (°C)	Water Temperatures	Rainfall (Millimetres)	Average UV Index
Brisbane	30.7	21.1	25 °C to 28 °C	179.8	11 (extreme)
Narragansett	28	18	20 °C to 24 °C	86.0	3.2 (moderate)

**Table 2:** Macroalgal species identified within the algal bloom/drift in Narragansett, Rhode Island, USA and Wynnum, Australia, including their morphological characteristics, habitat preferences, and the biotechnological applications of bioactive compounds isolated from these taxa.

Macroalgal Species	Features/Habitat	Biotechnological Use/MAAs [Ref]
Chlorophyta		
*Bryopsis* spp.	Filamentous green alga grows in tufted or mat-like manner, forming a dense mat of filaments	Biostimulants and biopesticides, drug discovery, anticancer [91]
*Codium fragile* subsp. *tomentosoides*	Edible green seaweed/attaches to a wide variety of hard substrates, including rocks, low intertidal and subtidal zones	Used in traditional Chinese medicine to treat enterobiasis, and due to its anti-inflammatory function and ability to improve the skin barrier, proposed for use in treating atopic dermatitis [88]
*Ulva* spp. (blade + tube morphologies)	Known as “sea lettuce”/free-floating or attached, numerous species	Use in human and animal nutrition, and biomaterials; improve water quality by absorbing excess nutrients and pollutants (e.g., heavy metals) [89]/MAAs
Phaeophyceae		
*Ascophyllum nodosum*	Known as rockweed/rocky coastlines, found in the northern Atlantic Ocean	Valuable compounds like polysaccharides (fucoidan, alginates), auxins, and phlorotannins [94]; biostimulants for plant growth and agricultural productivity [95]
*Fucus* spp.	Known as rockweed, edible seaweeds	Valuable bioactive compounds with antioxidant, anti-inflammatory, anti-tumour, anti-obesity, anticoagulant, and anti-diabetes [96]/MAAs
*Sargassum* spp.	Found in the subtidal region on semi-exposed shores [97]	Valuable source of nutrients and therapeutic compounds [98], antidiabetic, antioxidative, anti-fungal [99,100]
Rhodophyta		
*Champia parvula*	Found in intertidal pools, growing on sandy rocks, epiphytic (growing on other seaweeds)	Anticancer, antioxidant [101], antidiabetic [102]; anti-viral against the dengue mosquito vector for use as natural mosquitocidal agents [91,103]
*Chondrus crispus*	Known as Irish moss, edible seaweed/typically occurs intertidally and subtidally on rocky shores	Dietary supplement, anti-inflammatory and antioxidant, anticancer, antivenom (model species in scientific research) [84]
*Dasya baillouviana*	Found in tropical marine waters, the intertidal and shallow subtidal areas	Anti-grazing compounds due to bromophenols [104]
*Dasysiphonia japonica*	An invasive species/algal blooms	Compounds with antiviral and anti-inflammatory properties, decaying *D. japonica* caused mortality in juvenile-stage fish and larval bivalves/low MAAs [86]
*Gracilaria* spp.	Found from tropical to temperate waters, grow in intertidal and subtidal zones	Used as food, and in therapeutic purposes due to the unique composition of polysaccharides, pigments, and secondary metabolites [83]/MAAs: porphyra-334, shinorine, palythine, and palythenic acid [105,106]
*Grateloupia turuturu*	Found in protected shallow waters (e.g., the lower intertidal or upper subtidal zones), invasive in the North Atlantic Ocean	For developing new pharmaceuticals, antimicrobial, antioxidant and anti-inflammatory activities [73,107]/MAAs: shinorine, palythine and asterina-330 [74]
*Polyides rotundus*	Tolerate a wide temperature range; found in rocks in intertidal pools and shallow subtidal zones	Source of carrageenan, sulphated polysaccharides, used as a thickener, emulsifier, and stabiliser in foods [76,108]

Abbreviations: Mycosporine-Like Amino Acids (MAAs). Red algae—Rhodophyta; Green algae—Chlorophyta; brown algae—Phaeophyceae.

**Table 3:** Physicochemical properties for predicted antimicrobial peptides (AMPs) reported within the dbAMPs database (dbAMP) for algal bloom species *Chondrus crispus* and assessed using AMP Scanner Version 2 (i.e., APS v2), an open-source, deep learning-based tool designed for predicting and identifying AMPs using FASTA-formatted protein sequences.

dbAMP ID Residues Molecular Weight	Net Charge (+)	Aliphatic Index	pI	II	GRAVY	3D Structure of Proposed AMPs	Prediction Probability/CLASS
dbAMP_33036 29 2965.31	1	54.14	7.98	39.80	−0.479	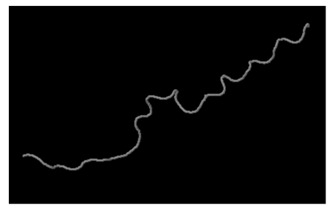	0.0385/ non-AMP
dbAMP_33037 31 3210.82	8	75.16	12.30	49.26	−0.297	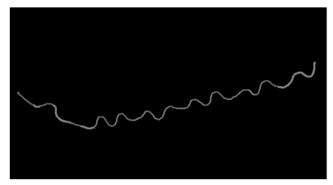	0.9913/ AMP
dbAMP_33038 30 3507.12	6	71.33	11.78	60.62	−0.700	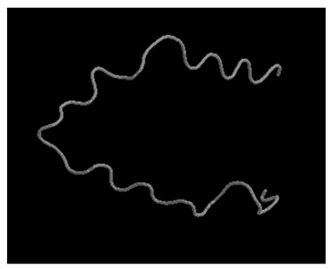	0.9997/ AMP
dbAMP_33039 45 5349.36	9	75.78	11.90	88.32	−0.291	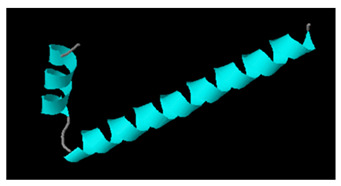	0.9997/ AMP
dbAMP_33040 40 4750.65	7.5	61	10.22	77.34	−0.660	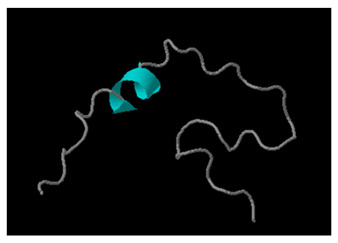	0.2497/ non-AMP
dbAMP_33041 26 2740.1	0.5	86.15	6.50	14.92	0.227	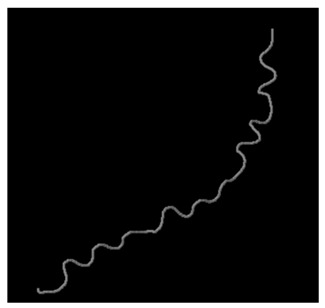	0.003/ non-AMP

Abbreviation: Theoretical isoelectric points (pI). The instability index (II). Grand average of hydropathicity (GRAVY). Note: Prediction probabilities > 0.5 = predicted AMP; <0.5 = predicted non-AMP.

## Data Availability

All the data are included within the article.
